# On the Effect of Sex on Prefrontal and Cerebellar Neurometabolites in Healthy Adults: An MRS Study

**DOI:** 10.3389/fnhum.2016.00367

**Published:** 2016-08-02

**Authors:** Dominique Endres, Ludger Tebartz van Elst, Bernd Feige, Stephan Backenecker, Kathrin Nickel, Anna Bubl, Thomas Lange, Irina Mader, Simon Maier, Evgeniy Perlov

**Affiliations:** ^1^Section for Experimental Neuropsychiatry, Department for Psychiatry and Psychotherapy, University Medical Center FreiburgFreiburg, Germany; ^2^Department for Psychiatry and Psychotherapy, Saarland University Medical CenterHomburg, Germany; ^3^Department for Radiology, Medical Physics, University Medical Center FreiburgFreiburg, Germany; ^4^Department of Neuroradiology, University Medical Center FreiburgFreiburg, Germany

**Keywords:** MR spectroscopy, sex, choline, creatine, cerebellum

## Abstract

In neuropsychiatric research, the aspects of sex have received increasing attention over the past decade. With regard to the neurometabolic differences in the prefrontal cortex and the cerebellum of both men and women, we performed a magnetic resonance spectroscopic (MRS) study of a large group of healthy subjects. For neurometabolic measurements, we used single-voxel proton MRS. The voxels of interest (VOI) were placed in the pregenual anterior cingulate cortex (pACC) and the left cerebellar hemisphere. Absolute quantification of creatine (Cre), total choline (t-Cho), glutamate and glutamine (Glx), N-acetylaspartate, and myo-inositol (mI) was performed. Thirty-three automatically matched ACCs and 31 cerebellar male–female pairs were statistically analyzed. We found no significant neurometabolic differences in the pACC region (Wilks' lambda: *p* = 0.657). In the left cerebellar region, we detected significant variations between the male and female groups (*p* = 0.001). Specifically, we detected significantly higher Cre (*p* = 0.005) and t-Cho (*p* = 0.000) levels in men. Additionally, males tended to have higher Glx and mI concentrations. This is the first study to report neurometabolic sex differences in the cerebellum. The effects of sexual hormones might have influenced our findings. Our data indicates the importance of adjusting for the confounding effects of sex in MRS studies.

## Introduction

In neuropsychiatric research, aspects of sex have received increasing attention over the past decade. There are interesting sex distribution ratios in populations with neurodevelopmental disorders such as attention deficit hyperactivity disorder (ADHD) and autism spectrum disorders (ASD), which are more common in boys than in girls (Biederman and Faraone, 2005; Tebartz van Elst et al., 2013). In adulthood, women are more frequently affected by depression and anxiety (Karger, 2014). Knowledge about the physiology of sex-related differences is the basis for understanding the pathophysiology of neuropsychiatric sex-related differences. Morphometric studies have shown that males' brains are 9–12% larger than females, although the relevance of this is unclear (Lenroot and Giedd, 2010). Connection studies have illustrated higher intra-lobe (within one hemisphere) neural communication in male brains and higher inter-lobe (between the left and right hemispheres) neural communication in female brains (Ingalhalikar et al., 2014). On a functional level, there are differences in the functional organization of language in the brain; during phonological tasks, brain activation in males is lateralized to the left frontal lobe, while female brains experience more diffuse activation involving the bilateral inferior frontal gyrus (Shaywitz et al., 1995).

### Sex vs. gender

In biopsychological research, the terms “sex” and “gender” are often used synonymously (Schuurs and Verheul, 1990). However, in a strict sense, “sex” refers to the biological state of being male or female according to an individual's genetic code, while “gender” focuses on the social role an individual plays in society, that is, whether a person feels and acts according to the expected roles of a female or male. We do not address gender issues in this sense, but rather, investigate the possible cerebral differences in the neurometabolite levels of healthy female and male individuals. Therefore, we use the terms “sex” or “sex-related” to report our findings, not to be confused with the colloquial use of “sex” for sexual intercourse, which is of no issue in this paper.

### Neurometabolite signals in females and males

Magnetic resonance spectroscopy (MRS) is a unique non-invasive method for measuring the homeostatic levels of various neurometabolites in the brain. Chemical shift imaging (CSI) allows researchers to analyze metabolite ratios in greater regions and perform secondary selection of voxels of interest (VOI). Single-voxel spectroscopy enables absolute quantification of neurometabolites in the predetermined VOI. The established proton MRS (1H-MRS) allows researchers to measure creatine (Cre) levels, the total choline (t-Cho) signal, the combined glutamate (Glu), and glutamine (Gln) signal (Glx), N-acetylaspertate (NAA), and myo-Inositol (mI) (Ross and Bluml, 2001). Cre serves a marker of brain energy metabolism. Due to its constancy over time, it is also used as a reference substance in metabolite ratios (Malhi et al., 2002). The t-Cho signal consists mainly of phosphorylcholine and glycerylphosphorylcholine, and it is a marker of cell membrane turnover (Gujar et al., 2005). Glu is a major excitatory neurotransmitter (Novotny et al., 2003). Gln is the precursor and stored form of Glu in astrocytes (Govindaraju et al., 2000). NAA is a marker of neuronal and axonal integrity. The mI signal is a glial marker and is part of the phosphatidylinositol second messenger system (Ross and Bluml, 2001).

Previous studies analyzing neurometabolic differences between the sexes have mostly found differences in the t-Cho and Glx levels of males and females. The t-Cho levels in the anterior cingulate cortex (ACC) of males were found to be higher than those of females (Hädel et al., 2013). However, the neurometabolites of the left cingulum showed no difference between sexes (Grachev and Apkarian, 2000). Two small studies found that cerebellar neurometabolites did not differ between sexes (Pouwels and Frahm, 1998; Safriel et al., 2005). Higher concentrations of Glu were found in left female hippocampi (Hädel et al., 2013). An age-related Glu decrease in the basal ganglia was found in males, but not females (Sailasuta et al., 2008; Chang et al., 2009). Table 1 provides an overview of other previous studies investigating our topic.

**Table 1:** **Previous MRS studies on healthy subjects**.

**Study**	**N (male/female)**	**Methods**	**Age**	**Localization**	**Findings**
Pouwels and Frahm, 1998	17M: 17F	2 T, SVS, 1H-MRS (STEAM)	18–30 years	Parietal white/gray matter	mI ↑in females parietal white matter Cre ↔, t-Cho ↔, Glu ↔, NAA ↔
				Frontal white/gray matter; occipital white/gray matter thalamus; vermis cerebelli, **left cerebellar hemisphere**	Cre ↔, t-Cho ↔, Glu ↔, NAA ↔, mI ↔
Grachev and Apkarian, 2000	19M: 19F	1.5 T, SVS, 1H-MRS (STEAM)	19–31 years	Left orbital frontal cortex	Glc ↑ in females
				Left sensorimotor cortex	NAA ↑ in females
				Dorsolateral prefrontal cortex	Lactate ↑ in males
				Left thalamus, **left cingulum**, left insula	t-Cho ↔, Glu ↔, NAA, mI ↔, Glc ↔, GABA ↔, lactate ↔
Zhong et al., 2002	22M: 17F	4 T, SVS, 1H-MRS (PRESS)	20–88 years	Frontal white matter	t-Cho ↑ in males, Cre ↔, NAA ↔, mI ↔
				Thalami, basal ganglia	Cre ↔, t-Cho ↔, NAA ↔, mI ↔
Pfleiderer et al., 2004	29M: 23F (DLPFC), 40M: 22F (ACC)	1.5 T, SVS, 1H-MRS (PRESS)	20–75 years	Left/right dorsolateral prefrontal cortex, **left anterior cingulate cortex**	Cre↔, t-Cho↔, Glx↔, NAA↔
					*NAA in the left dorsolateral prefrontal cortex and left anterior cingulate cortex positively correlated with IQ in women*.
Safriel et al., 2005	32M: 40F	1.5 T, SVS, 1H-MRS (PRESS)	20–44 years	Gray matter in the cortical frontal, parietal, temporal, and occipital lobe, basal ganglia, thalamus; subcortical white matter in the parietal and frontal lobes; pons, **cerebellum** (*all regions could not be studied in each subject*)	NAA/Cre ↔, t-Cho/Cre ↔ (in all 10 regions)
Sailasuta et al., 2008; Chang et al., 2009^*^	39M: 23F	3 T, SVS, 1H-MRS (PRESS)	21–71 years	Parietal gray matter	t-Cho ↑ in males, Cre ↔, Glu ↔, NAA ↔
				Basal ganglia	NA ↑ in males, age-dependent Glu ↓ in males, Cre ↔, t-Cho ↔, Glu ↔
				Frontal gray/white matter	Cre ↔, t-Cho ↔, Glu ↔, NA(A) ↔
Doelken et al., 2009	13M: 16F	3 T, multivoxel technique, 1H-MRS	Average age: 29 years	Hippocampi, basal ganglia, insula cortex, cingulum, and precuneus; gray and white matter from the frontal and parietal lobes	NAA ↔, Glx ↔, t-Cho ↔, mI ↔, Cre ↔
Ostojic et al., 2011	24M: 26F	1.5 T, multivoxel technique, 1H-MRS	30–58 years	White matter bilateral anterior, in the middle and in posterior regions; gray matter of the anterior, middle and posterior regions	Cho/Cre ↑and NAA/Cre ↑ in the right frontal parafalcine cortex, NAA/Cho ↔ (in all regions)
Hädel et al., 2013	59M: 59F	3 T, SVS, 1H-MRS (PRESS)	19–55 years	Left hippocampus	Glu ↑ in females Cre ↔, t-Cho ↔, NAA ↔
				**Anterior cingulate cortex**	t-Cho ↑in males, Cre ↔, Glu ↔, NAA ↔

* *Chang et al. (2009) reported corrected results from Sailasuta et al. (2008). Studies measuring the ACC or cerebellar regions are in bold type. Abbreviations: M, male; F, female; T, Tesla; SVS, single voxel spectroscopy; 1H-MRS, proton magnetic resonance spectroscopy; STEAM, stimulated echo acquisition method; PRESS, point-resolved spectroscopy; Cre, creatine; t-Cho, phosphorylcholine + glycerylphosphorylcholine; Glx, glutamate + glutamine; NAA, N-acetylaspartate; mI, myo-Inositol; NA, N-acetyl; Glc, glucose; IQ, intelligence quotient. ↑, increased; ↓, decreased; ↔, no metabolite differences between groups*.

### Rationale of our study

The aim of our study was to analyze neurometabolic sex-related differences in a large sample of healthy adult subjects. The study was part of a large clinical trial funded by the German Federal Ministry of Science and Education (ADHD-NET: 01GV0605, 01GV0606) (Tebartz van Elst et al., 2014; van Elst et al., 2014; Endres et al., 2015; Maier et al., 2015; Philipsen et al., 2015). In this project, we performed structural imaging (Riedel et al., 2014; Maier et al., 2015) and analyzed neurometabolic signals in the pACC and left cerebellum of adult patients with ADHD and ASD (Tebartz van Elst et al., 2014; van Elst et al., 2014; Endres et al., 2015). The voxels were selected based on our earlier finding that glutamatergic alterations occur in these regions (Perlov et al., 2007, 2009, 2010). The ACC is an important region for neuropsychiatric research because it integrates information from other areas of the brain into the fronto-striato-thalamo-frontal circuits (Bush et al., 2000; Tebartz van Elst and Perlov, 2013), therefore contributing to emotional self-control, focused problem solving, error detection, and adaptive responses to changing conditions (Allman et al., 2001). The cerebellum has attracted increasing attention in the field of neuropsychiatry in recent years. Current models view the cerebellum as “smoother” and as a place for internal models of mental functions (Schmahmann, 2004; Ito, 2008). As stated in our trial protocol, which is published on the Internet, we initially planned to analyze a control group of 50 subjects (Philipsen, 2008). During the data generation process, we decided to increase the size of the control group to better answer our fundamental research questions about the effects of sex and age on both regions. Overall, 119 healthy controls were eligible for the MRS study. After the selection procedure, we included 82 pACC and 78 cerebellar high-quality spectra of well-investigated healthy controls. Having measured such a large sample of control subjects, we were able to perform a strict matching procedure for age and IQ levels. Earlier studies found that men had higher t-Cho concentrations in the pACC and that there were no differences in the left cerebellar hemispheres of males and females (Pouwels and Frahm, 1998; Pfleiderer et al., 2004; Hädel et al., 2013). Because a limited amount of data was available from studies with less restrictive methods and different methodological approaches, our statistical analyses were exploratory.

## Participants and methods

Approval from the local ethics committee was obtained before starting the study (Faculty of Medicine, Freiburg University, 217/06). The study was part of a larger, government-funded project and was registered by Current Controlled Trials (ISRCTN54096201). Some data from this large multi-center study have already been published in previous papers (Endres et al., 2015; Maier et al., 2015; Philipsen et al., 2015). All participants gave written consent before undergoing scanning.

### Recruitment of subjects and matching procedure

Healthy participants were recruited via announcements on the campus of the University Medical Center Freiburg. Overall, 119 healthy controls were eligible for the MRS study. After performing a strict selection procedure, we were able to assess 82 pACC and 78 cerebellar spectra with a balanced ratio of males and females for the cerebellar VOI (39 males to 39 females) and a nearly balanced ratio for the pACC region (40 males to 42 females). The details of the selection procedure have already been published (Endres et al., 2015). Participants with current neurological or psychiatric diseases or who had consumed psychotropic drugs over an extended period of time were excluded. Participants with psychiatric axis I disorders were excluded based on the Mini International Neuropsychiatric Interview (Sheehan et al., 1998). Depressive symptoms were assessed using the Beck Depression Inventory (BDI) (Hautzinger, 2006), and cognitive deficits were assessed using the Conners Adult ADHD Rating Scale (CAARS) (Conners, 1999). BDI scores higher than 18 and CAARS t-scores higher than 65 were pre-defined as exclusion criteria. Premorbid verbal IQ was assessed using the multiple-choice vocabulary intelligence test (Lehrl et al., 1995). After our initial recruitment efforts, the male and female groups were not exactly matched in terms of the possibly influential factors of age (Kaiser et al., 2005) and premorbid verbal IQ (Jung et al., 1999). Therefore, we performed an automatic matching procedure for these factors (Kaller et al., 2014; Tebartz van Elst et al., 2014; van Elst et al., 2014), tolerating age differences of ≤5 years and IQ differences of ≤10 points between individual male–female pairs. This has resulted in optimal matching for 33 pACC and 31 cerebellum male and female controls.

### MRI data acquisition

**Figure 1:**
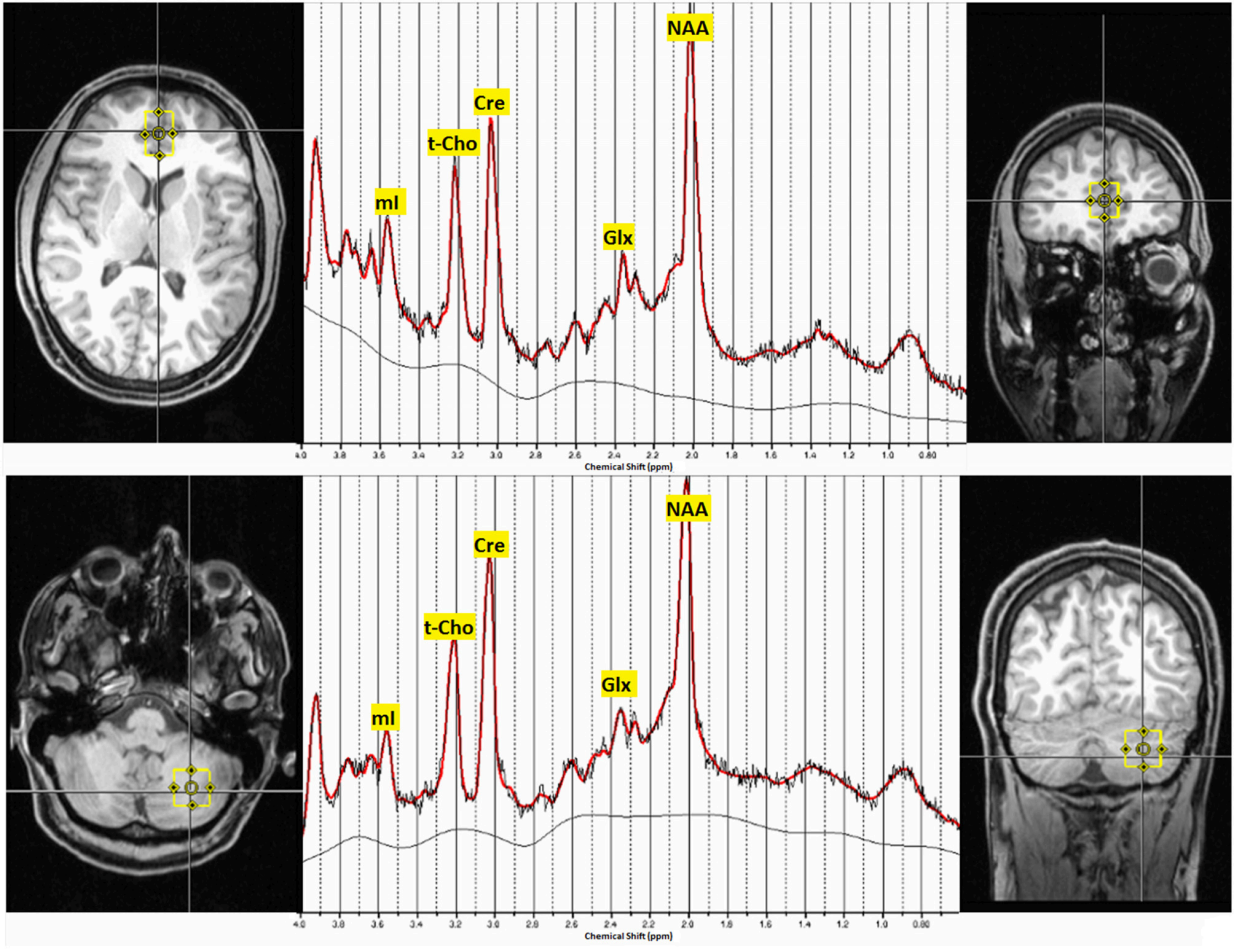
**Voxel localization in the pregenual ACC and the left cerebellar hemisphere and typical MRS-spectra**. Abbreviations: mI, myo-inositol; t-Cho, phosphocholine and glycerophosphorylcholine; Cre, creatine; Glx, glutamate and glutamine; NAA, N-acetylaspartate; ppm, parts per million.

All measurements were obtained using a 3 Tesla whole-body scanner (Siemens MAGNETOM Trio, a TIM System; Erlangen, Germany) with a 12-channel head coil. First, a T1-weighted three-dimensional imaging data set was recorded using a magnetization-prepared rapid acquisition gradient echo sequence with the following parameters: field of view = 256 × 256 mm^2^, repetition time = 2200 ms, echo time = 4.11 ms, flip angle = 12°, and voxel size = 1 × 1 × 1 mm^3^. For spectroscopic measurements, the VOIs were located in the pACC (16 × 25 × 20 mm) and the left cerebellar hemisphere (20 × 20 × 20 mm) (Figure 1). For 1H MRS, a point-resolved spectroscopy sequence with a repetition time of 3000 ms and an echo time of 30 ms (number of averages = 96) was used. For absolute quantification of metabolites, we acquired a non-water-suppressed reference spectrum.

### Spectroscopic analysis

MRS analysis was performed following a methodical procedure developed in earlier studies (Tebartz van Elst et al., 2014; van Elst et al., 2014; Endres et al., 2015). The well-known linear combination of the model spectra algorithm was applied for the investigator-independent spectral analysis (Provencher, 1993, 2001), while the absolute quantification of the neurometabolites was estimated using an internal water signal reference (Helms, 2008). Only those metabolites with Cramér–Rao lower bounds of <20% for the main metabolites were included in the statistical analyses (http://s-provencher.com/pub/LCModel/manual/manual.pdf). Each VOI was segmented into gray matter (GM), white matter (WM), and cerebrospinal fluid (CSF) using the unified segmentation approach developed by Ashburner and Friston (2005), which was implemented using Statistical Parametric Mapping, Version 8 (http://www.fil.ion.ucl.ac.uk/spm/software/spm8/). The metabolic concentrations in each voxel were corrected for the partial volumes of the GM, WM, and CSF based on the segmentation processes.

### Statistical analysis

All statistical analyses were performed using Statistical Package for the Social Sciences, Version 22. Group comparisons for parametric variables (age, IQ, nicotine consumption, and psychometric scores) were performed through independent sample *t*-tests. A *p* < 0.05 served as the criterion for significance. Metabolite concentrations in the male and female groups were compared using a multivariate analysis of covariance with a general linear model. Sex was set as a fixed factor in this analysis, while the metabolite concentrations of Cre, t-Cho, Glx, NAA, and mI were set as dependent variables. To investigate the overall effect of all five metabolite concentrations on the groups, a multivariate Wilks' lambda test was performed. The level of significance for overall and single-group comparisons was corrected for multiple tests using the Bonferroni approach. We measured two regions, so a *p* < 0.025 was chosen as the criterion for significance. To test whether there was a dimensional relationship between metabolite signals that differ between groups and factors such as IQ, subclinical depressiveness, ADHD symptoms, and nicotine consumption, we performed a correlation analysis of these variables using an exploratory Pearson correlation coefficient. A *p* < 0.025 was chosen as the criterion for significance in the correlation analyses because we measured two regions.

## Results

### Demographic and psychometric information

The demographic and psychometric data are summarized in Table 2. Overall, the psychometric scores measuring the depressive symptoms (BDI), attention impairment (CAARS), and hyperactivity (CAARS) were not significantly different between the sexes. The possibly confounding variable of nicotine consumption (cigarettes per day) was balanced between the groups.

**Table 2:** **Demographic and psychometric data**.

	**Male (*n* = 33)**	**Female (*n* = 33)**	***p*-value**	**Male (*n* = 31)**	**Female (*n* = 31)**	***p*-value**
	**Mean ± SD**	**Mean ± SD**		**Mean ± SD**	**Mean ± SD**	
	**Pregenual anterior cigulate cortex**			**Left cerebellar hemisphere**		
Age	35.76 ± 9.523 (range: 23–58)	35.48 ± 9.884 (range: 22–58)	0.909	36.48 ± 9.359 (range: 23–58)	36.29 ± 9.399 (range: 22–58)	0.936
IQ (Lehrl et al., 1995)	120.64 ± 16.695	120.67 ± 16.635	0.994	120.19 ± 16.672	120.61 ± 16.233	0.920
BDI (Hautzinger, 2006)	2.3045 ± 3.23478	2.3061 ± 3.59217	0.999	2.4210 ± 3.30358	2.3581 ± 3.67440	0.944
CAARS-Total (Conners, 1999)	38.18 ± 9.831	34.42 ± 6.906	0.077	38.03 ± 9.820	33.97 ± 5.523	0.050
Nicotine	1.0909 ± 2.87623	1.6364 ± 4.51953	0.561	1.1613 ± 2.95631	1.2903 ± 3.95132	0.885

*Abbreviations: SD, standard deviation; IQ, intelligence quotient (measured by MWTB = “Mehrfachwahl-Wortschatz-Intelligenz-Test”); BDI, Beck depression inventory; CAARS, Conners' Adult ADHD Rating Scales. Nicotine: Consumption in cigarettes per day*.

### MRS results

**Figure 2:**
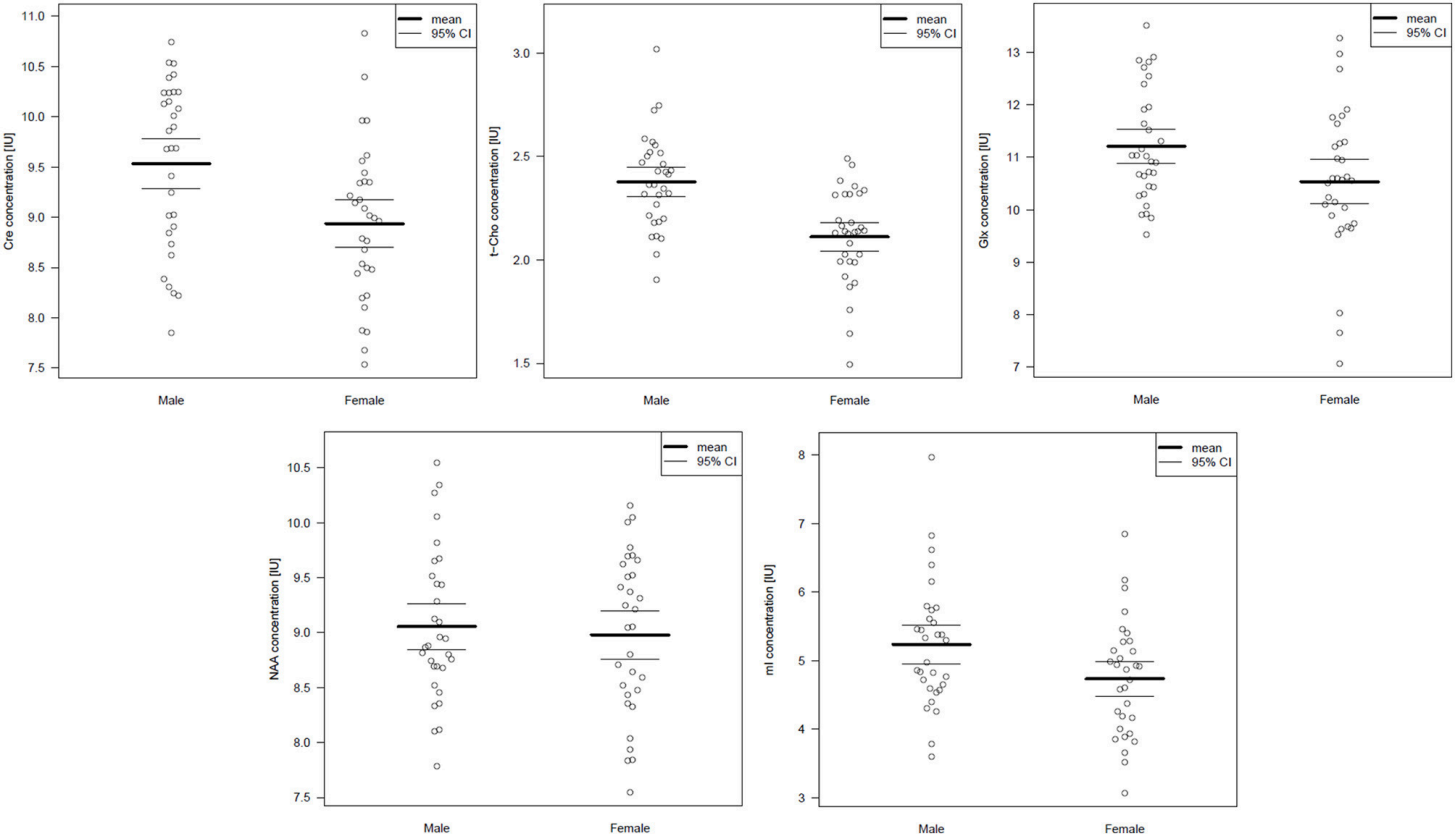
**Cerebellar metabolite concentrations presented as scatterplots**. Abbreviations: Cre, creatine; t- Cho, phosphorylcholine + glycerylphosphorylcholine; Glx, glutamate + glutamine; NAA, N-acetylaspartate; mI, myo-Inositol.

Table 3 summarizes the spectroscopic results. Upon comparing the male and female groups, we found no significant differences in the pACC VOI (Wilks' lambda: *p* = 0.657). In the left cerebellar region, we found significant differences between the groups (Wilks' lambda: *p* = 0.001). In the male group, Cre and t-Cho levels were significantly increased. In addition, men tended to have higher Glx and mI concentrations, although this was no longer significant after performing the Bonferroni correction (Figure 2). The voxel composition (GM, WM, and CSF) did not differ significantly between the groups.

**Table 3:** **Spectroscopic findings from the pregenual anterior cingulate cortex and the cerebellum**.

	**Male (*n* = 33)**	**Female (*n* = 33)**	**Statistics MANCOVA**	**Male (*n* = 31)**	**Female (*n* = 31)**	**Statistics MANCOVA**
	**Mean ± SD**	**Mean ± SD**	(Wilks'-lambda: *p* = 0.675)	**Mean ± SD**	**Mean ± SD**	(Wilks'-lambda: *p* = 0.001)
	**Pregenual anterior cingulate cortex**			**Left cerebellar hemisphere**		
**Cre**	8.7765 ± 1.35235	8.6416 ± 1.23539	*F* = 0.179, *p* = 0.674	9.5325 ± 0.82719	8.9359 ± 0.77770	*F* = 8.559, *p* = 0.005^*^
**t-Cho**	2.3039 ± 0.38403	2.1825 ± 0.40183	*F* = 1.573, *p* = 0.214	2.3769 ± 0.22968	2.1114 ± 0.22831	*F* = 20.834, *p* = 0.000^*^
**Glx**	15.9069 ± 2.27305	15.6276 ± 1.95646	*F* = 0.286, *p* = 0.594	11.2101 ± 1.06483	10.5301 ± 1.38934	*F* = 4.678, *p* = 0.035^**^
**NAA**	11.2808 ± 1.31831	11.2864 ± 1.20675	*F* = 0.000, *p* = 0.986	9.0551 ± 0.68609	8.9793 ± 0.72651	*F* = 0.179, *p* = 0.674
**mI**	6.0527 ± 0.94888	6.0200 ± 0.96456	*F* = 0.019, *p* = 0.890	5.2363 ± 0.92073	4.7326 ± 0.84443	*F* = 5.038, *p* = 0.029^**^

*Abbreviations: SD, standard deviation; Cre, creatine; t-Cho, phosphorylcholine + glycerylphosphorylcholine; Glx, glutamate + glutamine; NAA, N-acetylaspartate; mI, myo-Inositol*.

* *Significant after Bonferroni correction*;

** *not significant after Bonferroni correction*.

### Dimensional analysis

We found no correlation between the cerebellar metabolite signals that differed between groups (Cre and t-Cho signals) and psychometric scores in the control groups. In the pACC of male subjects, we found a significant correlation between t-Cho signals and BDI scores (*r* = 0.423; *p* = 0.014; *N* = 33). No significant correlations were found in the female group.

## Discussion

The main finding of this study is that Cre and t-Cho signals in the left cerebellar hemisphere are higher in males than in females. We found no differences between sexes in the pACC.

### Sample selection and spectroscopic imaging

The healthy subjects were thoroughly tested. Through a battery of psychometric tests, psychiatric axis I and II disorders were excluded. A history of neurological disorders, drug consumption, or neurological or psychiatric medications also led to exclusion. We tried to create age- and IQ-matched male and female groups using an automatic matching procedure (Kaller et al., 2014). Methodologically, the well-established single voxel 1H-MRS was used. Cerebellar brain size differs between the sexes (Raz et al., 1998, 2001), as does the distribution of Cre and t-Cho concentrations in the WM and GM (Ross and Bluml, 2001). Therefore, we corrected for the GM, WM, and CSF content of each measured VOI. In doing so, we excluded false positive results caused by different VOI compositions.

### Comparison to previous studies

As noted in the introduction, three studies have investigated the cingulate region, and two studies have examined the cerebellum with respect to sex effects. Hädel et al. (2013) examined 118 healthy controls in the ACC and, like us, found no difference between sexes in terms of Cre, Glx, NAA, and mI concentrations, although they found increased anterior cingulate t-Cho concentrations in males. Another study also found higher anterior cingulate t-Cho-signals in males (Pfleiderer et al., 2004). In the third study investigating the cingulate region, no metabolic differences between sexes were found in the left cingulum (Grachev and Apkarian, 2000). In our study, the t-Cho concentration in the pACC showed a slight tendency to be higher in the male group (2.3039 in males vs. 2.1825 in females; *p* = 0.214). However, this finding was not significant, similar to the findings of Pfleiderer et al. (2004). The difference in the findings of the present study and those of Hädel et al. might be due to different matching procedures. Hädel et al. (2013) did not create age- and IQ-matched groups; although they used age as a covariate, they did not correct for education or intelligence. A recent study, however, has shown that t-Cho is correlated with IQ (Jung et al., 1999). Thus, IQ should be corrected for, as it is a confounding effect.

To our knowledge, cerebellar neurometabolites were analyzed in only two previous studies. The first study found no differences between sexes in a sample of 34 healthy controls (17 males and 17 females) using a different single-voxel spectroscopy sequence (stimulated echo acquisition mode) (Pouwels and Frahm, 1998). The second study comparing small groups of 10 male and 10 female subjects showed no alterations in NAA/Cre and t-Cho/Cre ratios in either group (Safriel et al., 2005). Increased t-Cho concentrations have been found in various regions of males' brains, including the parietal GM, frontal WM, and ACC (Zhong et al., 2002; Chang et al., 2009; Hädel et al., 2013). In our exploratory study, we detected a correlation between t-Cho scores and depression scores in the male group. Therefore, the t-Cho alterations found in earlier studies might have been influenced by depression. To our knowledge, differences in Cre concentrations have not been described in earlier studies.

### Neurochemical perspective

Neurochemically, we found significantly higher Cre and t-Cho concentrations in males' cerebella. The Cre peak, which includes creatine and phosphocreatine, reflects total cellular Cre stores. Regulation takes place via (1) enzyme equilibrium between Cre and phosphocreatine, which plays a central role in energetic adenosine triphosphate synthesis; (2) the biosynthetic pathway through liver and kidney enzymes, where Cre is synthesized; and (3) osmotic forces with increased hyperosmolar states and decreased hypoosmolar states (Ross and Bluml, 2001). Despite this complex regulation, Cre signals remain relatively constant over time and therefore are frequently used as a reference substance in metabolite ratios (Malhi et al., 2002). In this respect, our finding that Cre alterations in the cerebellum depend on one's sex is crucial for MRS research in cerebellar regions because it could explain the strong influence of sex on metabolite ratios (with Cre as the denominator).

The t-Cho signal consists of phosphorylcholine and glycerylphosphorylcholine and, to a smaller degree, the neurotransmitter acetylcholine. In 1H-MRS, the t-Cho signal serves as a marker of membrane turnover because it includes precursors and degradation products of cell membrane phospholipids (Hajek and Dezortova, 2008). Many focal, inflammatory, and hereditary diseases lead to an increase in the t-Cho signal. Cell membrane turnover is an energy-dependent process and thus might be linked to Cre energy metabolism.

### Pathophysiological interpretation

What are the reasons for different t-Cho and Cre concentrations in the left cerebellar hemisphere between sexes?

Obviously, the influence of the sex hormones might explain cerebellar neurometabolic variations between the sexes (Rasgon et al., 2001; Rapkin et al., 2011). Ovarian steroid hormones (i.e., primary excitatory estrogens and primary inhibiting progesterone) are widespread in the brain and have modulating effects on brain function (Majewska, 1987; Rasgon et al., 2001). In an earlier study on female subjects, t-Cho/Cre ratios in the parietal WM were found to significantly differ from the mid-follicular stage to the late luteal phase of the menstrual cycle; however, Cre concentrations and the role of the cerebellum were not analyzed (Rasgon et al., 2001). Neurometabolic studies using positron emission tomography with [18F]fluorodeoxyglucose showed menstrual cycle-dependent changes in the cerebellum. There was also an increase in cerebellar activity from the follicular phase to the late luteal phase of the menstrual cycle (Rapkin et al., 2011). In patients with premenstrual dysphoric disorder, these changes were correlated with mood changes (Rapkin et al., 2011). Based on these findings, one might speculate that our finding that cerebellar neurometabolic profiles differ between sexes indicates not structural but ovarian steroid-related changes in neuronal function.

### Role of the cerebellum

The cerebellum has received increased attention in neuropsychological and neuropsychiatric research in the past decade. Schmahmann and Sherman defined cerebellar cognitive affective syndrome, which is characterized by disturbances in executive functions, impaired spatial cognition, personality and affect changes, and linguistic difficulties (Schmahmann and Sherman, 1998). The dysmetria of thought theory and the internal modeling machine hypothesis provide explanations for these findings. The dysmetria of thought theory describes the cerebellum as a “smoother” vehicle for motor and mental functions (Schmahmann, 2004), while the internal modeling machine hypothesis views mental activities as controlled by internal models in the cerebellum (Ito, 2008). One might speculate that our finding that cerebellar neurometabolites differ between sexes could be associated with disease distribution (e.g., ADHD, ASD, depression) among the sexes. Findings of this nature are of interest to the public, and further studies are needed to gain a more precise understanding of this topic. Future studies focusing on the differing roles of the cerebellum in males and females should employ hormonal testing, multimodal structural and neurometabolic imaging, and parallel motor and neuropsychological testing.

## Conclusion

In our study, we found no significant differences in the pACCs and distinct differences in the left cerebellar neurometabolites of males and females. The finding that Cre is altered in the cerebellum is important for MRS research because it could explain the strong influence of sex on Cre-dependent metabolite ratios. MRS studies should analyze sex-balanced groups in a standardized manner or should otherwise correct for sex. Further multimodal studies analyzing the role of the cerebellum in males and females should be performed.

## Author contributions

DE and LTvE conducted the data analysis and wrote the paper. DE, LTvE, SM, and EP organized the study. BF and SB supported the data analysis. TL and IM gave technical support. All authors were crucially involved in the theoretical discussion and performing of the manuscript. All authors read and approved the final version of the manuscript.

## Funding

Parts of the study were funded by the German Federal Ministry of Science and Education (BMBF; ADHD-NET: 01GV0605, 01GV0606).

### Conflict of interest statement

DE, BF, SB, KN, AB, TL, SM, and EP declare that the research was conducted in the absence of any commercial or financial relationships that could be construed as a potential conflict of interest. LTvE: Advisory boards, lectures, or travel grants within the last three years: Eli Lilly, Janssen-Cilag, Novartis, Shire, UCB, GSK, Servier, Janssen, and Cyberonics. IM: Lecture fees from Bracco Imaging Deutschland GmbH, Germany; Roche Pharma AG, Germany; UCB Pharma GmbH, Germany.
